# A New Double Salt, the Sulphite of the Protoxide of Platinum and the Sulphite of Soda, Discovered and Investigated by Messrs. A. Litton and Schnederman

**Published:** 1843-02

**Authors:** 


THE WESTERN JOURNAL.

Vol. VII.—No. II.

LOUISVILLE, FEBRUARY 1, 1843.

We publish the following communication with great pleasure, since it affords us an opportunity of introducing to the profession one of the most accomplished young chemists in our country. The writer, Mr. Litton, of Nashville, has just returned from Europe, where he spent several years in the arduous prosecution of chemical studies in the laboratories of Wöhler, Liebig and other great masters. He has come home full of zeal in his favorite science, with ample knowledge and skill as a practical chemist, and only wants a situation to be useful to his country. Y.

*A new Double Salt, the Sulphite of the Protoxide of Platinum and the Sulphite of Soda, discovered and investigated by Messrs A. Litton and Schnederman.*

This compound is formed, when sulphurous acid gas is conducted into a solution in water of the chloride of platinum, and this fluid is saturated by the carbonate of potash. By this method is produced a very voluminous and almost colourless precipitate, which, as investigation showed, is a double salt of the sulphite of the protoxide of platinum with the sulphite of soda.

This body is, when dried, an amorphous, white powder; when moist it has a yellowish tinge, which is the deeper, the more the solution is concentrated, from which it is precipitated. In cold water it is slightly soluble. The solution is colourless, and neutral, and leaves the salt, upon evaporation, as a white varnish-like substance. In warm water it is more soluble, since the hot saturated solution becomes, upon cooling, troubled and untransparent: however, the quantity dissolved is always small. In alcohol it is insoluble. From its solution in water it is precipitated, by the chloride of sodium and several other salts, as a fleecy powder, and the compound thus thrown down is perfectly white.

A remarkable property of this salt is that, when dissolved in water, the presence of platinum is not indicated by its common tests. The solution remains unchanged upon the addition of sulphuretted hydrogen and hydrosulphuret of ammonia. When, however, an acid is at the same time added, by which the salt may be decomposed, the fluid, at the ordinary temperature, is slowly colored; by heating it becomes immediately a brownish red, and the sulphuret of platinum soon falls down. This precipitate is soluble when heated in solutions of the sulphurets of the alkalies. By the caustic alkalies the salt is not decomposed.

By acids, even when they are diluted, it is decomposed, and, with the development of sulphurous acid, dissolved. The solution in hydro-chloric acid, gives, upon evaporation, crystals of the chloride of sodium, and, upon the addition of ammonia, a green crystallinish precipitate of the platina proto-chloride of the hydro-chlorate of ammonia. The solution in sulphuric acid gives, when evaporated, crystals of the sulphate of soda, and presents the dark color of the sulphate of the protoxide of platinum. By a certain degree of concentration, metallic platinum falls down from this solution, a property which was found to belong to the sulphate of the protoxide of platinum prepared for this purpose. The solution in nitric acid assumes a deep brownish-red color, from which, by the addition of sal ammoniac, no precipitate is obtained; if, however, the fluid be then evaporated almost to dryness, the platino-chloride of the hydrochlorate of ammonia is found in abundance. The brownish-red color seems to arise from the formation of the sulphate of the deutoxide of platinum, with which the reaction towards sal ammoniac agrees. In a solution of the cyanuret of potassium, the double salt is very soluble, from which, by evaporating, crystals of the platino-cyanuret of potassium may be obtained.

When the salt is exposed to a temperature of 200° (Centigrade), it loses completely the water which it holds chemically united. Heated to 240° it suffers no further change, but exposed to a still greater heat it undergoes decomposition. This is completely effected by continued exposure to a red heat, whereby a quantity of the sulphate, and sulphite of soda, with metallic platinum, remains behind.

In order to determine the quantity of soda and platinum, the salt was mixed with sal ammoniac and heated red hot. The residue, which consisted of chloride of sodium and metallic platinum, was thoroughly washed with distilled water, and the platinum weighed: while the water, after the addition of sulphuric acid, was evaporated to dryness, in order to determine the soda as a sulphate. To determine the quantity of sulphurous acid, the salt was diffused through water, into which chlorine was conducted, and the sulphuric acid thus obtained by oxidizing the sulphurous, was precipitated by the chloride of barium.

Of the salt dried by 200° (Cen.)

1st Exp. 1.850 grs. gave 1.190 sulphate of soda=0.5214 soda; and 0.543 platinum—0.587 protoxide of platinum.

2d Exp. 1.108 grs. gave 0.328 platinum=0.3546 protoxide of platinum.

3d Exp. 1.488 grs. gave 0.954 sulphate of soda=0.418 soda.

4th Exp. 0.867 grs. gave 1.234 sulphate of baryta=0.3395 sulphurous acid.

5th Exp. 0.874 grs. gave 1.249 sulphate of baryta=0.3436 sulphurous acid.

These numbers give for the composition of the salt, when free from water, the following formula:

$$(3Na.O\ SO_2 + Pl.O\ SO_2)$$

According to which in one hundred parts are contained,

| | *Calculated.* | *Experiments.* | | | | |
|---|---|---|---|---|---|---|
| | | 1 | 2 | 3 | 4 | 5 |
| Soda, | 28.53 | 28.18 | | 28.09 | | |
| Protoxide of platinum, | 32.44 | 31.73 | 32.00 | | | |
| Sulphurous acid, | 39.03 | | | | 39.16 | 39.32 |

The salt, when dried at 100° (Cen.), lost upon being heated to 200° according to three different experiments, 3.90, 4.28, 4.16 per cent. of water; from which, for the composition of the salt containing water, is obtained the following formula:

$$2\ (3Na.O\ SO_2 + Pl.O\ SO_2) + 3HO.$$

according to which formula, the water should amount to 3.94.

When protoxide of platinum is diffused through water and sulphurous acid conducted therein, it is gradually, although difficultly, dissolved with a greenish brown color, and from this solution, by the carbonate of soda, the above described salt may be obtained.

If the salt is dissolved in only so much diluted sulphuric or hydrochloric acid as is requisite for its solution, and the fluid then evaporated by a gentle heat, in proportion as the sulphurous acid escapes, a yellow powder falls down, which is also a compound of the sulphite of the protoxide of platinum, with the sulphite of soda, but containing a smaller quantity of the latter than the above salt.

After being well washed and dried by 100° (Cen.)

1 Exp. 0.884 grs. of this salt gave 0.306 sulphate of soda=0.1341 soda; and 0.410 platinum=0.4432 protoxide of platinum.

2 Exp. 0.443 grs. gave 0.487 sulphate of baryta=0.1339 sulphurous acid.

These numbers give for the salt this formula—:

$$(Na.O\ SO_2 + Pl.O\ SO_2) + HO.,$$

according to which the salt contains in 100 parts:

| | *Calculated.* | *Experiment.* 1 | 2 |
|---|---|---|---|
| Soda, | 14.81 | 15.17 | |
| Protoxide of platinum, | 50.53 | 50.13 | |
| Sulphurous acid, | 30.40 | | 30.22 |
| Water, | 4.26 | | |

The quantity of water was not directly found.

This salt is difficult to be obtained in a large quantity, because it is tolerably soluble in water, and after being well washed in order to free it from all impurities, but comparatively little remains upon the filter. When dissolved in water, its solution acts feebly acid; it is not precipitated by chloride of sodium, but its solution exhibits otherwise most of the properties of the first described salt.

Above I have sent you a translation of the researches made by a young German chemist and myself in the laboratory of Professor Wöhler, at Gottingen. Should the paper not be too different from such as find admittance into your journal, will you please publish it.

Yours truly,

Nashville Dec. 16, 1842. A. LITTON.

